# Sex-dependent and compartment-specific macrophage accumulation associates with glomerular injury in BTBR *ob/ob* mice

**DOI:** 10.1007/s00424-026-03195-8

**Published:** 2026-07-28

**Authors:** Mariana Charleaux de Ponte, Victoria Mel Dussan Angulo, Leticia Barreto da Silva, Karina Thieme

**Affiliations:** Laboratory of Cellular and Molecular Bases of Renal Physiology, Department of Physiology and Biophysics, Institute of Biomedical Sciences, University of Sao Paulo, Sao Paulo, SP Brazil

**Keywords:** Diabetic kidney disease, BTBR *ob/ob*, Glomerular dysfunction, Glomerular inflammation, Sex differences

## Abstract

Type 2 diabetes mellitus (T2DM) is a metabolic disorder characterized by chronic hyperglycemia and represents a growing global health burden. One of its major complications is diabetic kidney disease (DKD), a progressive condition characterized by declining kidney function accompanied by structural alterations in tissue. Among the multiple pathways involved in DKD progression, inflammation has emerged as a key contributor. In parallel, increasing evidence suggests that biological sex influences disease progression; however, whether and how sex modulates inflammatory mechanisms driving DKD progression remains incompletely understood. In this study, we investigated the interplay between inflammation and biological sex in DKD using the BTBR *ob/ob* model, which closely recapitulates the human disease. Obese diabetic mice exhibited significant albuminuria regardless of sex; however, podocyte-associated proteins displayed sex-dependent molecular regulation. At the inflammatory level, no changes were detected in whole-kidney analyses based on the selected markers, but a compartment-specific response was observed, characterized by increased macrophage infiltration and upregulation of *Ccl2* (MCP-1) gene expression in the glomerular compartment, especially in males. Additionally, the tubular compartment exhibited distinct sex- and metabolism-dependent inflammatory gene expression patterns. Together, these findings indicate that inflammation in this model is spatially compartmentalized and also differentially regulated according to sex. This integrated perspective may contribute to a better understanding of DKD progression and support the development of more precise and targeted therapeutic strategies.

**Graphical Abstract:**

**Figure:**
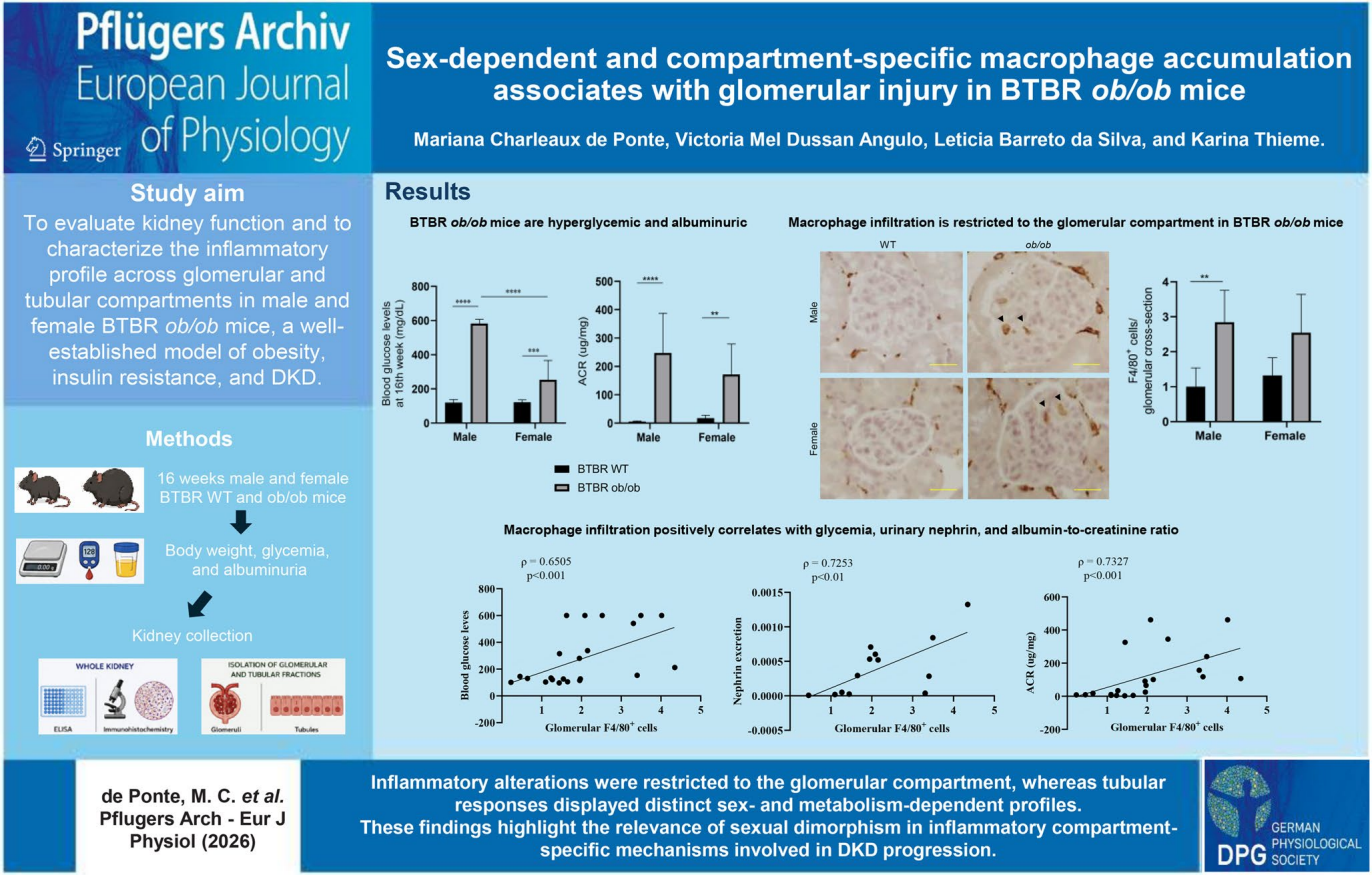

**Supplementary Information:**

The online version contains supplementary material available at 10.1007/s00424-026-03195-8.

## Introduction

Diabetes mellitus is a metabolic disorder characterized by chronic hyperglycemia [1]. Globally, it is estimated that approximately 90–95% of cases correspond to type 2 diabetes mellitus (T2DM) [2], which is primarily marked by impaired insulin action, a pancreatic hormone responsible for promoting glucose uptake in peripheral tissues and thus modulating energy metabolism [3]. Prolonged hyperglycemia is associated with pathological changes affecting multiple tissues and organs, including blood vessels, eyes, and kidneys [4–6]. Approximately 20–50% of individuals with T2DM develop diabetic kidney disease (DKD) [7, 8], one of the most common complications in T2DM and a leading cause of chronic kidney disease (CKD) and end-stage renal disease (ESRD). Clinically, DKD is characterized by persistent albuminuria and reduced glomerular filtration rate (GFR), reflecting progressive impairment of kidney function [9].

With the rising prevalence of obesity, T2DM, and DKD, there has been growing interest in investigating the influence of biological sex on these disorders. Women are more prone to developing obesity; however, the prevalence of T2DM is frequently higher in men [10], reaching up to 61% in some populations [11]. Although men with T2DM generally have a higher risk of ESRD and mortality [12], some studies report that women, with poor HbA1c and/or LDL-cholesterol control may exhibit a faster decline in kidney function [13, 14]. In addition, the pattern of DKD differs between sexes, with men more frequently presenting an albuminuric phenotype [15], whereas women tend to exhibit functional alterations characterized by reduced GFR [14]. These observations suggest that the development of DKD may be influenced by biological sex, which can modulate pathological pathways involved in disease progression.

Among the pathological pathways involved in DKD progression, inflammation has emerged as a key contributor [16]. Chronic hyperglycemia induces the overproduction of proinflammatory cytokines and chemokines in renal tissue, leading to immune cell recruitment and activation of inflammatory responses, ultimately resulting in impaired kidney function [17]. When sustained, inflammation contributes to renal structural alterations, including mesangial expansion, glomerular basement membrane thickening, and extracellular matrix accumulation, which are hallmarks of fibrosis and progressive kidney injury [18]. Circulating levels of inflammatory proteins, particularly those of the tumor necrosis factor-α (TNF-α) pathway, including its receptors TNFR1 and TNFR2, have been associated with the 10-year risk of ESRD, indicating that inflammatory proteins may serve as effective biomarkers of DKD [19]. In addition, circulating and urinary levels of the inflammatory chemokine MCP-1 (CCL2) are associated with early stages of DKD, supporting its potential as a biomarker of disease onset [20, 21]. Thus, inflammation is widely recognized as an important contributor to DKD progression.

Given that men and women may exhibit distinct patterns of DKD progression and that inflammation is widely recognized as an important contributor to this process, it remains incompletely understood whether sexual dimorphism influences proinflammatory mechanisms and, consequently, renal impairment in DKD. It has been suggested that diabetic male mice display higher levels of proinflammatory cytokines and increased immune cell infiltration compared to females, which has been associated with worse renal outcomes [22]. In contrast, evidence from human studies is inconsistent, with some reports indicating no effect of sex on DKD progression [23], whereas others describe sex-specific differences in systemic inflammatory profiles [24]. In addition, most studies assess inflammation at the systemic or whole-kidney level, potentially overlooking compartment-specific alterations within the kidney. Therefore, the present study aimed to evaluate kidney function and to characterize the inflammatory profile across glomerular and tubular compartments in male and female BTBR *ob/ob* mice, a well-established model of obesity, insulin resistance, and DKD that closely recapitulates key pathophysiological features observed in patients. This study seeks to contribute to a better understanding of sex-related differences in DKD progression and to support the development of more targeted therapeutic strategies.

## Materials and methods

### Animals

Heterozygous black and tan brachyury obese mice (BTBR/ob; BTBR.Cg-Lepob/WiscJ; stock no. 004824) were obtained from Jackson Laboratory (Bar Harbor, ME, USA) and housed at the experimental facility of the Department of Pharmacology, Institute of Biomedical Sciences, University of São Paulo. Offspring were genotyped, and male and female obese mice (BTBR *ob/ob*) and their respective wild-type littermates (BTBR WT) were used at 16 weeks of age. Animals were maintained under standard laboratory conditions, with ad libitum access to food and water and a controlled light–dark cycle. All experimental procedures were approved by the Animal Care and Use Ethics Committee of the Institute of Biomedical Sciences, University of São Paulo (protocol numbers 5348280918 and 7358100320).

### Metabolic parameters

Body weight gain and blood glucose levels were measured every two weeks from 8 to 16 weeks of age. Blood glucose was determined after a 2-hour fast using a glucometer (Accu-Chek^®^ Performa, Roche Diagnostics, São Paulo, SP, Brazil). At the end of the protocol, kidneys were removed, decapsulated, and weighed, and the kidney-to-body weight ratio was calculated.

### Albuminuria

Urinary albumin was measured using the AssayMax™ Mouse Albumin ELISA Kit (Assaypro, St. Charles, MO, USA), according to the manufacturer’s instructions. Urinary creatinine levels were determined by an enzymatic colorimetric assay using a commercial kit (Labtest, Lagoa Santa, MG, Brazil). The albumin-to-creatinine ratio (ACR) was calculated for each sample. Urine samples were also subjected to 10% SDS-PAGE and subsequently stained with silver using the SilverQuest™ Silver Staining Kit (Sigma-Aldrich, St. Louis, MO, USA). A 5% bovine serum albumin (BSA) solution was used as a marker and positive control.

### Separation of glomerular- and tubular-enriched fractions

Glomerular- and tubular-enriched fractions were obtained from whole kidney tissue as previously described [25], with modifications. Briefly, approximately 0.20–0.25 g of kidney tissue was isolated, and the medulla was carefully removed. The remaining cortical tissue was finely minced using a sterile blade in 4 mL of RPMI medium containing 0.325 U/mL of Liberase™ TL (Roche, Basel, Switzerland). The tissue suspension was incubated in a water bath at 37 °C for 20 min, with mechanical dissociation performed every 5 min using an 18-gauge needle to optimize enzymatic digestion. Liberase activity was terminated by adding an equal volume of RPMI supplemented with 10% fetal bovine serum (FBS). The suspension was centrifuged at 300 g for 5 min at 4 °C, the supernatant was discarded, and the pellet was resuspended in 5 mL of Hank’s Balanced Salt Solution (HBSS). The suspension was filtered through a 100 μm cell strainer (Corning, New York, USA). The original tube was rinsed with 5 mL of HBSS, and the content was passed through the same strainer to maximize tissue recovery. The filtrate was subsequently filtered through a 70 μm cell strainer. The resulting suspension (~ 45 mL) was then filtered through a 40 μm cell strainer. The filtrate, containing the tubular-enriched fraction, was collected for subsequent centrifugation. The 40 μm strainer was then transferred to a new tube and washed with 45 mL of HBSS to remove residual tubular fragments. The strainer was then transferred to a Petri dish and washed with 25 mL of HBSS to recovery the remaining structures. The suspension was incubated for 2 min to allow differential adhesion, in which tubular fragments adhered to the dish surface while glomeruli remained in suspension. The supernatant (~ 25 mL), enriched in glomeruli, was passed again through a new 40 μm strainer, and the filtered was discarded. The remaining glomeruli were washed with an additional 25mL of HBSS and incubated for 2 min in a new Petri dish. The resultant supernatant was then transferred to a 50 mL conical tube containing 0.01% polyvinylpyrrolidone (PVP) to prevent glomerular adhesion to the tubules walls. Both glomerular- and tubular-enriched fractions were centrifuged at 1000 g for 5 min at 4 °C. The supernatants were discarded, and the pellets resuspended in 500 µL of HBSS and transferred to a microtube. After centrifugation at 5000 g for 2 min at 4 °C, the supernatants were removed, and the pellets were immediately frozen on dry ice and stored at −80 °C until further analysis. All plastic tubes and cell strainers were pre-treated with 10% bovine serum albumin (BSA) prior to use to prevent tissue adhesion and minimize sample loss.

### Gene expression by real time PCR

The glomerular- and tubular-enriched fractions were submitted to RNA isolation. RNA from the tubular-enriched fractions was isolated using *Trizol* reagent (Invitrogen, Waltham, MA, USA), followed by purification with isopropanol. RNA from glomerular-enriched fractions was isolated and purified using RNeasy Micro Kit (Qiagen, Venlo, Limburg, Netherlands), according to the manufacturer’s instructions. RNA was quantified, and 2000 ng from tubular-enriched fractions and 200 ng from glomerular-enriched fractions were used for cDNA synthesis. cDNA was synthesized using the High-Capacity RNA-to-cDNA Kit (Applied Biosystems, Foster City, CA, USA), according to the manufacturer’s instructions. The cDNA and *TaqMan* probes were used for real time PCR on the StepOne system (Applied Biosystems). The data were analyzed using the 2^ΔΔCt^ method and were presented as fold change relative to the male BTBR WT group. *Ppia* gene expression was used as the reference for normalization in tubular-enriched samples, whereas in glomerular-enriched samples, normalization was performed using the arithmetic mean of the endogenous reference genes *Ppia* and *Actb*. The *TaqMan* probes used were: *Ppia* (Mm02342430_g1), *Actb* (Mm00607939-s1) *Nphs1* (Mm01176615_g1), *Nphs2* (Mm01292252_m1), *Tnf* (Mm99999068_m1), *Tnfrsf1a* (Mm00441883_g1), *Tnfrsf1b* (Mm00441889_m1), *Ccl2* (Mm00441242_m1), *Ccr2* (Mm99999051_gH), *Nfkb1* (Mm00476361_m1), *Lcn2* (Mm01324470_m1), and *Hvcr1* (Mm00506686_m1).

### Quantification of kidney proteins by ELISA

Kidneys were removed, weighed, and homogenized in PBS supplemented with protease inhibitor cocktail (P8340, Sigma-Aldrich, St. Louis, MO, USA) at a final concentration of 1% (v/v), using a tissue-to-buffer ratio of 1:5 (w/v). Renal homogenates were used for total protein quantification (Bradford method; Biorad, Hercules, CA, USA) and for the determination of TNF, TNFR1, TNFR2 and MCP-1 protein levels using commercially available colorimetric ELISA kits, according to the manufacturer’s instructions (Elabscience, Houston, TX, USA). The presented data were normalized to total protein concentration.

### Protein expression by immunofluorescence

Kidney sections were deparaffinized, rehydrated, and subjected to antigen retrieval using EDTA buffer (1 mM EDTA, 0.05% Tween 20, pH 8.0). Sections were then blocked for 1 h (Protein Block, DAKO, Agilent Technologies Inc., Santa Clara, CA, USA) and incubated overnight at 4 °C with rabbit anti-Podocin primary antibody (1:200, P0372, Sigma-Aldrich). The following day, sections were incubated with Rhodamine Red™-X goat anti-rabbit secondary antibody (1:200, Jackson ImmunoResearch Laboratories, PA, USA) and then the nuclei were counterstained with 4′,6-diamidino-2-phenylindole (DAPI) (1:20000, Sigma-Aldrich). Images were acquired using NIS-Elements software (Nikon, Minato, Tokyo, Japan) on an Eclipse 80i microscope and podocin signal was quantified as mean gray value of the red channel within glomerular regions using ImageJ.

### Protein expression by immunohistochemistry

Kidney sections were processed as described above for immunofluorescence, including deparaffinization, rehydration, antigen retrieval, and blocking. Sections were then incubated overnight at 4 °C with primary antibodies against F4/80 (1:500, Cell Signaling Technology Inc., Danvers, MA, USA) and TNFR2 (1:300, Invitrogen). On the next day, the slices were incubated with the HRP-conjugated secondary antibody (DAKO Agilent Technologies), and staining was developed using 3,3′-diaminobenzidine (DAB). Sections were subsequently counterstained with hematoxylin. F4/80 staining in whole kidney sections was quantified using ImageJ. Images were color-deconvolved using the H DAB vector, and a consistent color threshold was applied. The percentage of positive area relative to total tissue area was used for comparisons between groups. For glomerular analysis, regions of interest corresponding to glomerular area were manually defined. After color deconvolution and thresholding, F4/80-positive staining within glomeruli was quantified by particle analysis using a size threshold of 400–∞ pixels². TNFR2 staining in glomeruli was quantified using the same procedure, except that the particle size threshold was set to 0–∞ pixels².

### Statistical analysis

The data were analyzed using a two-way ANOVA, considering both metabolic condition and sex factors. Tukey’s post-hoc test was performed subsequently. Results are presented as mean ± SD. P values < 0.05 were considered statistically significant. Spearman’s correlation analysis was performed to assess the association between the number of glomerular F4/80-positive cells and blood glucose levels, nephrin excretion, and albumin-to-creatinine ratio (ACR) and a p value < 0.05 was considered statistically significant.

Outlier detection and exclusion was performed using the ROUT (Robust Regression and Outlier Removal) method implemented in GraphPad Prism. Some differences in sample size among experiments were due to the availability and suitability of biological samples for each specific assay. The sample sizes are specified in the figure legends.

## Results

### BTBR *ob/ob* mice are obese and hyperglycemic, with males exhibiting higher blood glucose levels than females

**Fig. 1:**
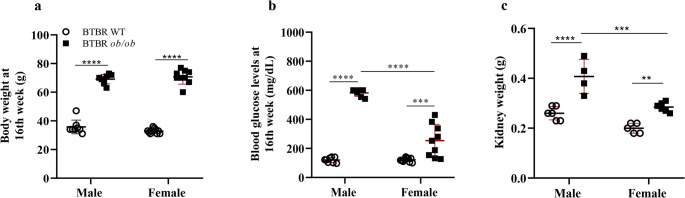
Metabolic profile in BTBR *ob/ob* model. Body weight, g (**A**), blood glucose levels, mg/dL (**B**) and kidney weight, g (**C**) in BTBR WT and *ob/ob* mice with 16 weeks of age. P values of two-way ANOVA followed by Tukey’s multiple comparisons test are as follows: **p < 0.01, ***p < 0.001, and ****p < 0.0001. The values are the mean ± SD (*n* = 4–10)

Figure 1 describes the metabolic characterization of the BTBR *ob/ob* model. At 16 weeks of age, male and female BTBR *ob/ob* mice presented increased body weight and blood glucose levels when compared with the respective WT groups (Fig. 1a and b). However, the elevation in blood glucose levels was more pronounced in male mice in comparison to females (Fig. 1b). Diabetic and obese mice also showed an increased kidney weight, with a greater increase observed in males compared to females (Fig. 1c). Together, these results confirm the obese and hyperglycemic phenotype of the BTBR *ob/ob* model and demonstrate that male mice develop more severe hyperglycemia than females despite comparable body weight.

### Sex modulates podocyte gene and protein expression in albuminuric BTBR *ob/ob* mice

**Fig. 2:**
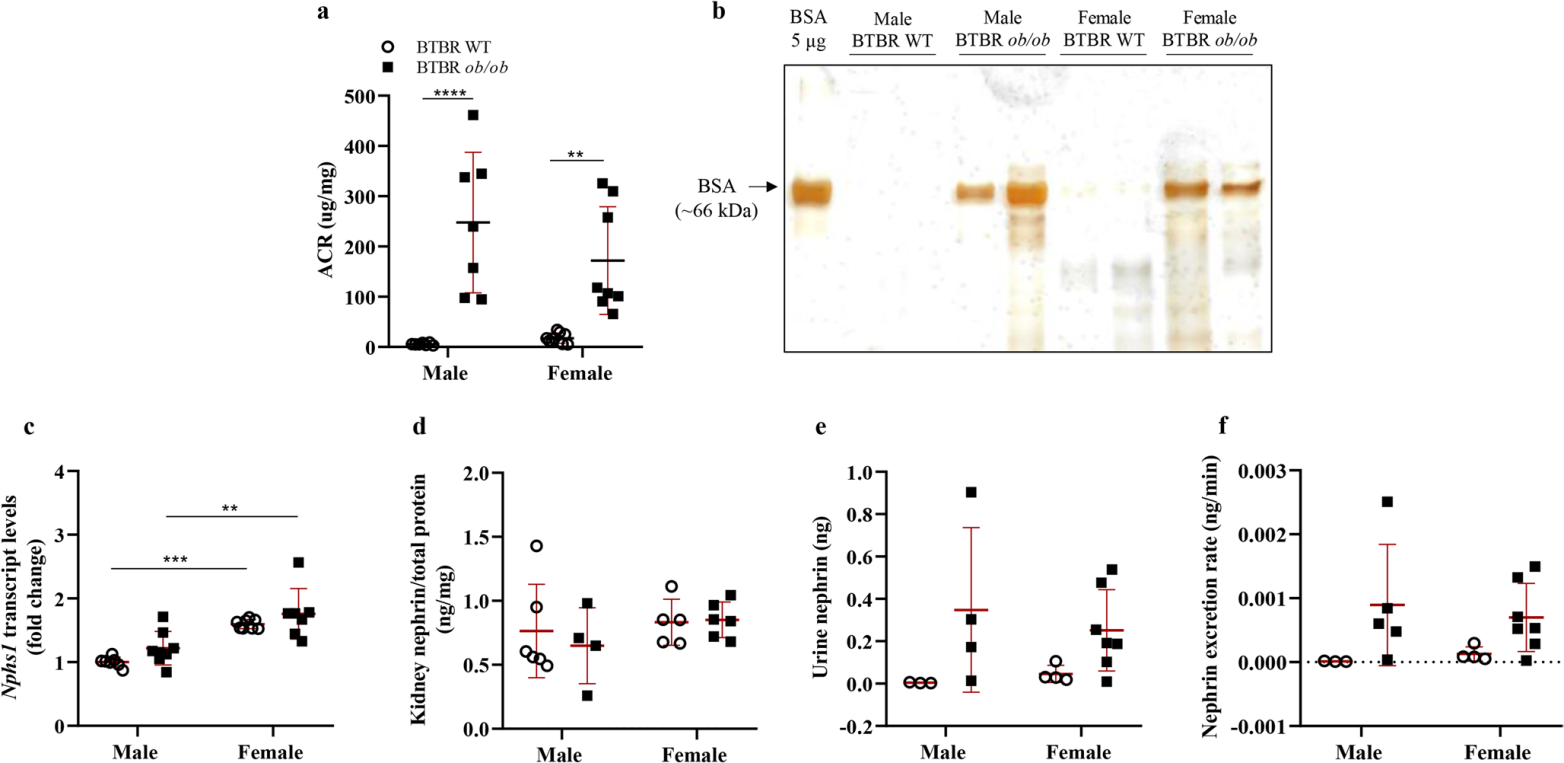
Kidney function and nephrin gene expression, protein levels, and urinary excretion in BTBR *ob/ob* model. Albumin-to-creatinine ratio (ACR) in urine, µg/mg (**A**); silver-stained polyacrylamide gel showing urinary albumin levels (**B**); *Nphs1* (nephrin) transcript levels, expressed as fold change (**C**); nephrin levels normalized to total protein in renal homogenates, ng/mg (**D**); urinary nephrin levels, ng (**E**); and nephrin excretion rate, ng/min (**F**). P values of two-way ANOVA followed by Tukey’s multiple comparisons test are as follows: **p < 0.01, ***p < 0.001, and ****p < 0.0001. The values are the mean ± SD (*n* = 3–9). ACR: albumin-to-creatinine ratio; BSA: albumin

**Fig. 3:**
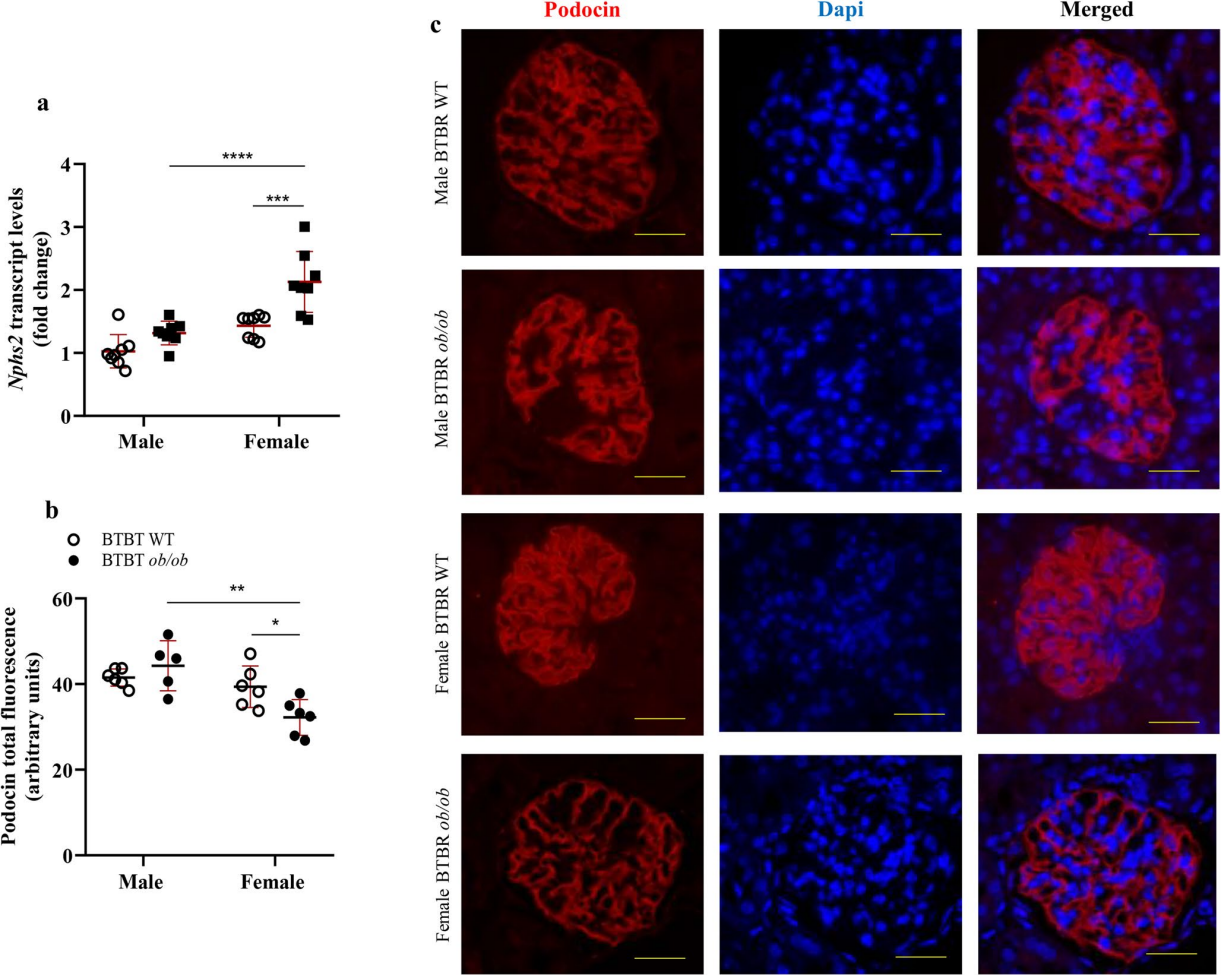
Gene and protein expression of podocin in BTBR *ob/ob* model. *Nphs2* (podocin) transcript levels, expressed as fold change (**A**); podocin quantification by immunofluorescence, expressed by arbitrary units (**B**); and representative immunofluorescence images showing podocin (red), DAPI (blue), and merged images. (**C**). Immunofluorescence images were acquired using NIS-Elements software coupled to an Eclipse 80i microscope with a 40× objective (excitation wavelength: 524 nm). Scale bars = 50 μm. P values of two-way ANOVA followed by Tukey’s multiple comparisons test are as follows: *p < 0.05, **p < 0.01, ***p < 0.001, and ****p < 0.0001. The values are the mean ± SD (*n* = 5–8)

Kidney function was assessed by measuring urinary albumin levels and by analyzing gene and protein expression of podocyte markers, given the association between podocyte dysfunction and albuminuria. As show in Fig. 2a, obese and diabetic mice presented elevated albumin-to-creatinine ratio (ACR) in comparison to the respective WT controls, regardless of sex. Silver staining of SDS–PAGE gel confirmed increased urinary albumin levels in BTBR *ob/ob* mice in relation to the WT groups (Fig. 2b). Nephrin gene expression (*Nphs1*) was increased in females compared to males, independent of the metabolic condition (Fig. 2c). However, no significant differences were observed in the nephrin-to-total protein ratio in kidney tissue, urinary nephrin levels, or nephrin excretion rate (Fig. 2d **– **f). Similarly, BTBR *ob/ob* females exhibited increased podocin gene expression (*Nphs2*) and reduced protein levels compared to BTBR *ob/ob* males and WT females (Fig. 3a **- **c). Taken together, these results confirm the presence of albuminuria in the BTBR *ob/ob* model and suggest sex-dependent modulation of gene and protein expression of podocyte markers, as well as differences in mRNA-protein coupling in obese females.

### Inflammatory markers at the whole-kidney level were not increased in obese and diabetic mice

**Fig. 4:**
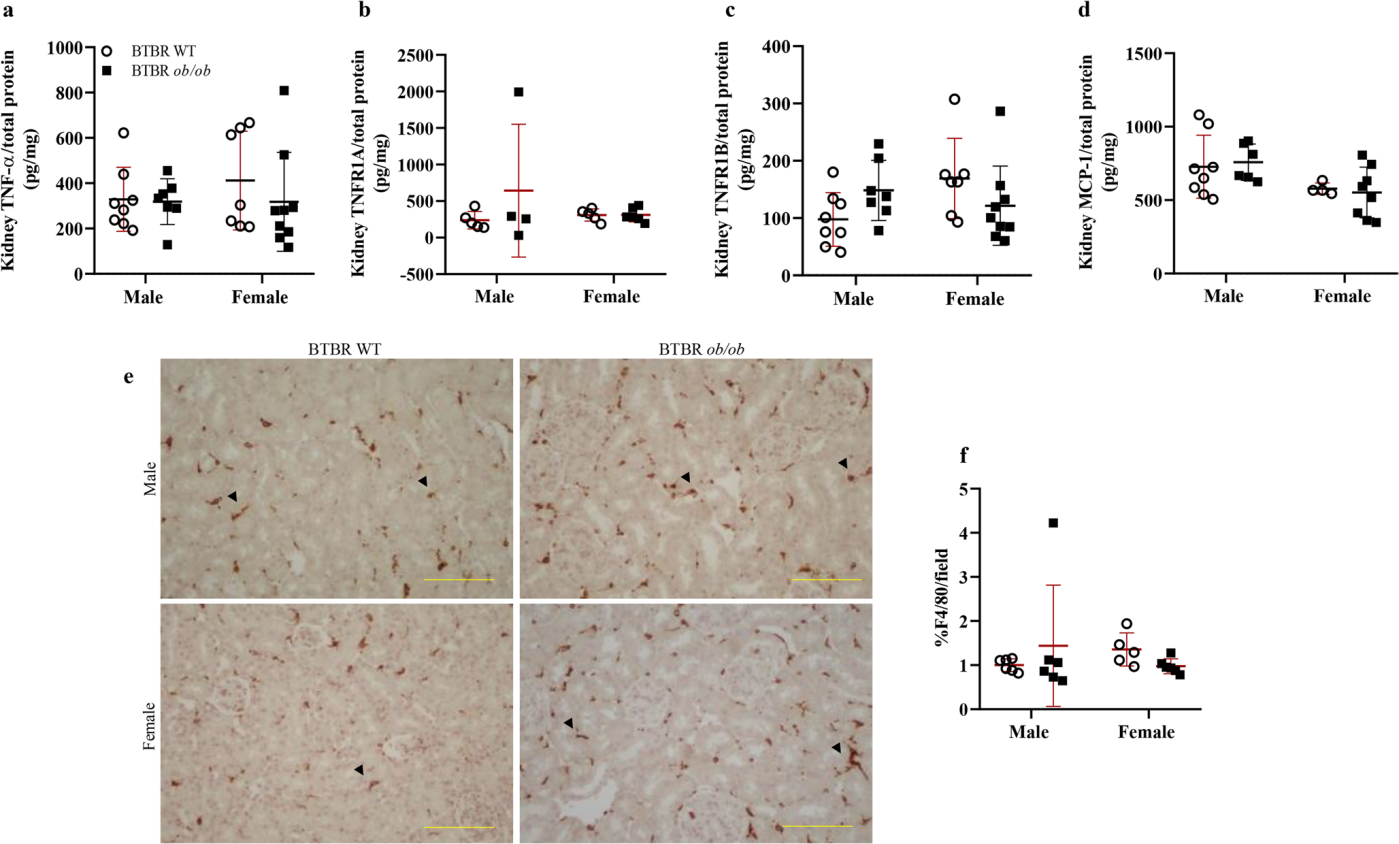
Inflammatory profile in whole-kidney tissue in the BTBR *ob/ob* model. Levels of TNF-α (**A**), TNFR1A (**B**), TNFR1B (**C**), and MCP-1 (**D**) in renal homogenates, normalized to total protein levels, pg/mg. Representative immunohistochemistry images showing F4/80 staining (**E**), and quantification of F4/80 expression in whole-kidney tissue, % to the field (**F**). Immunohistochemistry images were acquired using NIS-Elements software with an Eclipse 80i microscope equipped with a 20× objective. Scale bars = 50 μm. Data are presented as mean ± SD (*n* = 4–9)

The inflammatory profile of renal tissue was evaluated in the BTBR *ob/ob* model. No differences were observed in TNF-α, TNFR1A, TNFR1B, or MCP-1 levels in renal tissue at either 8 weeks (Supplementary Fig. 1) or 16 weeks of age (Fig. 4a **– **d), regardless of sex or metabolic condition. In addition, immunohistochemical staining for F4/80 was performed to assess macrophage infiltration. As shown in Fig. 4e and f, BTBR *ob/ob* mice did not exhibit increased F4/80 staining in whole kidney tissue. Together, these findings indicate that, at the whole-tissue level, the BTBR *ob/ob* model does not show evidence of kidney inflammation.

Supplementary Fig. 1: Inflammatory protein levels in renal homogenates of 8-week-old BTBR *ob/ob* mice. Levels of TNF-α (A), TNFR1A (B), TNFR1B (C), and MCP-1 (D) in renal homogenates, normalized to total protein levels, pg/mg. Data are presented as mean ± SD (*n* = 8–12). 

### Macrophage infiltration is restricted to the glomerular compartment in BTBR *ob/ob* mice

**Fig. 5:**
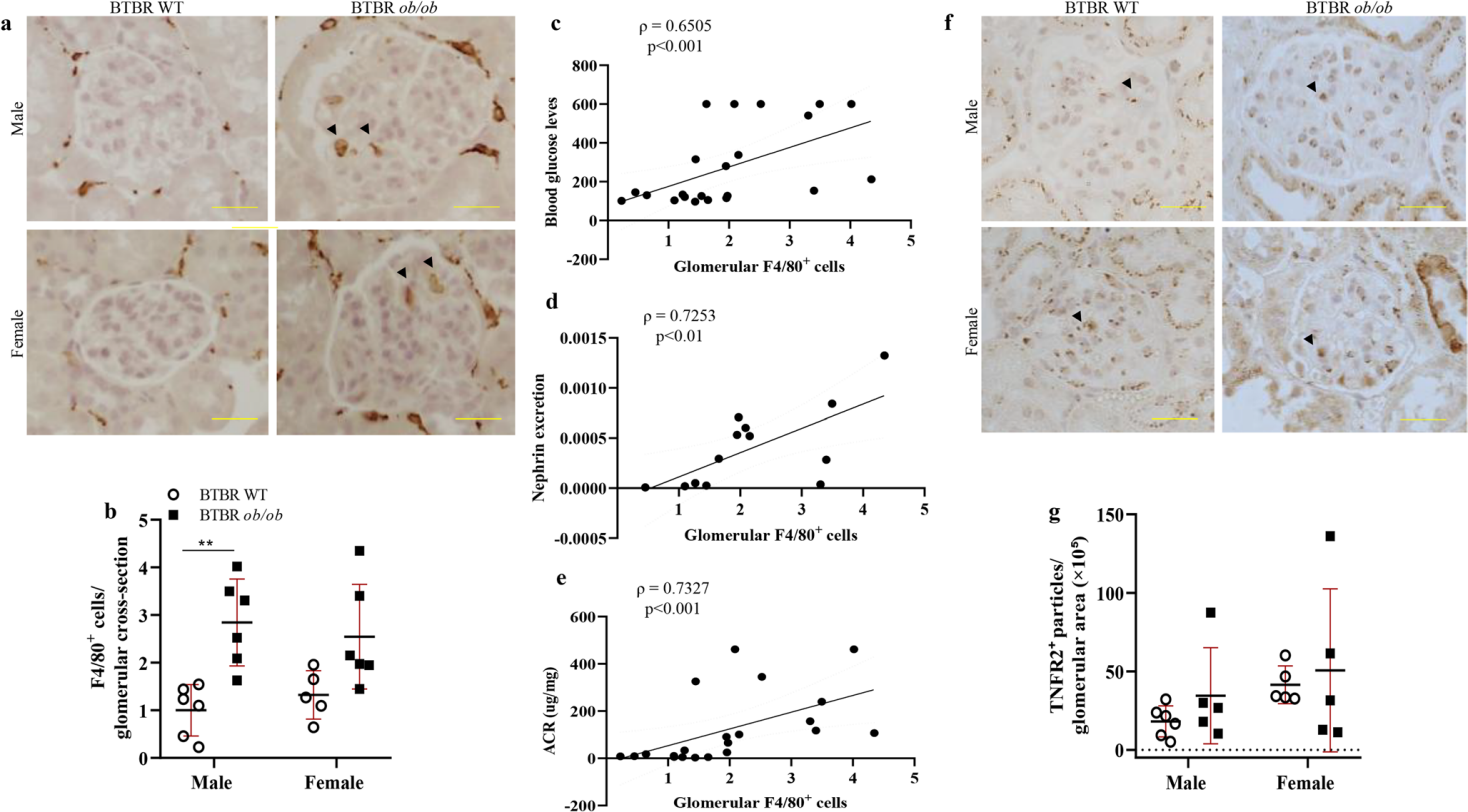
Inflammatory profile in the glomerular compartment in the BTBR *ob/ob* model. Representative immunohistochemistry images showing F4/80 staining (**A**), and quantification of F4/80 expression in the glomerular compartment, number of positive cells per glomerular cross-section (**B**). Images **C-E** represent the correlation between glomerular F4/80^+^ cells and blood glucose levels, nephrin excretion, and ACR, respectively. Representative immunohistochemistry images showing TNFR2 staining (**F**), and quantification of TNFR2 expression in the glomerular compartment, positive particles per glomerular cross-section (x10^5^) (**G**). Immunohistochemistry images were acquired using NIS-Elements software with an Eclipse 80i microscope equipped with a 40× objective. Scale bars = 50 μm. P values of two-way ANOVA followed by Tukey’s multiple comparisons test are as follows: **p < 0.01. Data are presented as mean ± SD (*n* = 5–6). Spearman correlation analyses were performed with experimental n ranging from 13 to 23

Animal models of diabetes and obesity indicate that the glomerulus is an early target of inflammation. Thus, although no inflammation was detected at the whole-kidney level, analyses were extended to the glomerular compartment. BTBR *ob/ob* male mice exhibited an increased number of F4/80-positive cells per glomerular cross-section compared to their respective WT controls (Fig. 5a and b). In addition, the increased macrophage infiltration was positively correlated with blood glucose levels (Fig. 5c), urinary nephrin excretion (Fig. 5d), and the ACR (Fig. 5e). Given the increased glomerular infiltration of macrophages observed in diabetic mice, TNFR2 expression was further evaluated in this compartment. No difference in TNFR2 expression were detected in the glomeruli of BTBR WT and *ob/ob* mice (Fig. 5g). These findings indicate that early glomerular macrophage accumulation may be driven by inflammatory mechanisms other than TNF-α signaling. Moreover, glomerular macrophage infiltration positively correlates with hyperglycemia and albuminuria, suggesting an association between glomerular inflammation and impairment of kidney function.

### Inflammatory and injury markers gene expression in renal tubules is differentially regulated by sex and metabolism

**Fig. 6:**
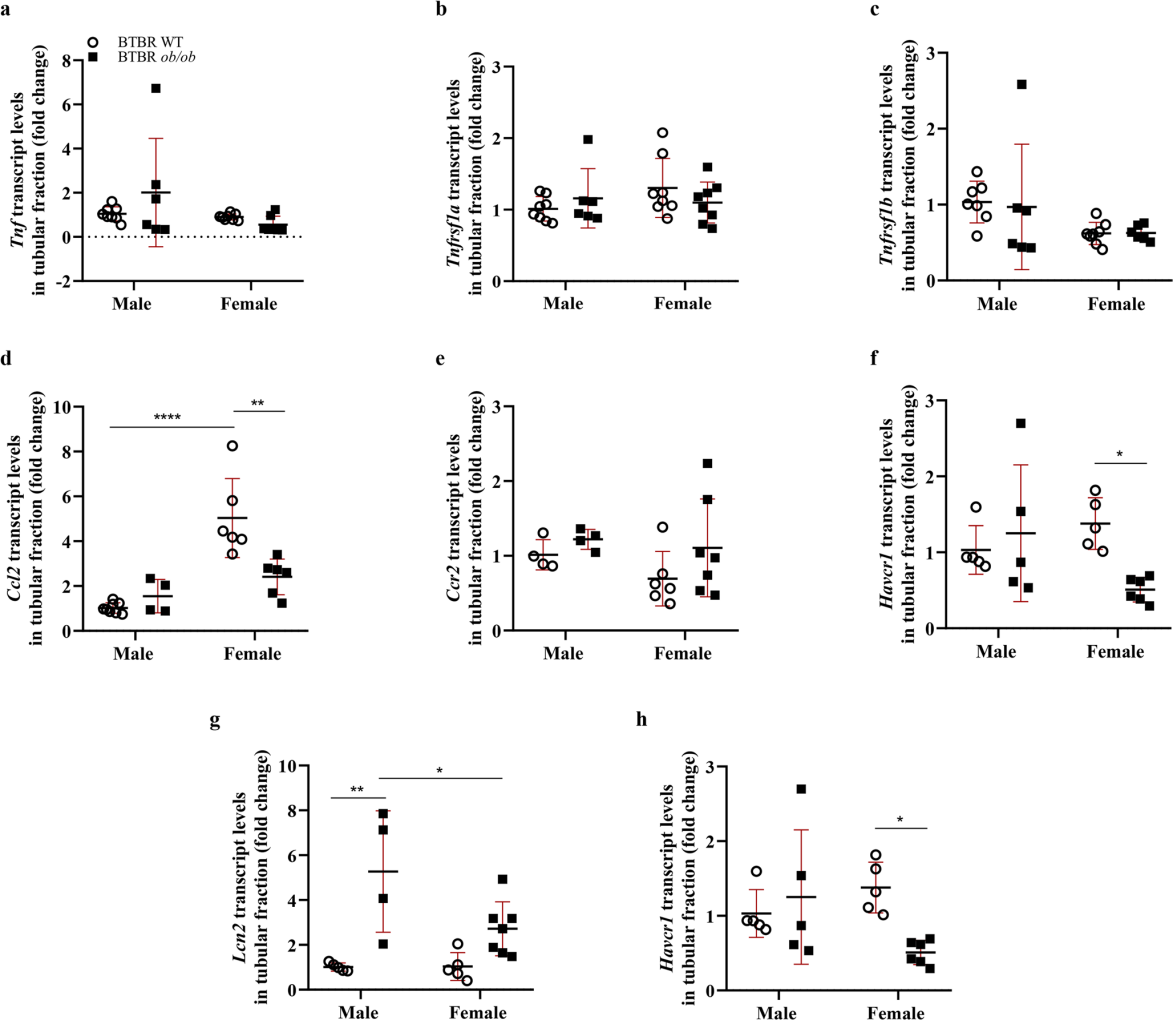
Inflammatory and injury markers gene expression profile in the tubular-enriched fraction of the BTBR ***ob/ob*** model. Images **A–F** show tubular transcript levels of *Tnf* (TNF-α), *Tnfrsf1a* (TNFR1), *Tnfrsf1b* (TNFR2), *Ccl2* (MCP-1), *Ccr2* (MCP-1 receptor), and *Nfkb1* (NFKB1), respectively, all expressed as fold change. P values of two-way ANOVA followed by Tukey’s multiple comparisons test are as follows: *p < 0.05, **p < 0.01, and ****p < 0.001. Data are presented as mean ± SD (*n* = 4–8)

Given the increased macrophage infiltration observed in the glomerular compartment, inflammatory gene expression was analyzed separately in tubular and glomerular compartments. To this end, tubular- and glomerular-enriched fractions were isolated. Analysis of tubular-enriched fractions showed no differences in the transcript levels of *Tnf*, *Tnfrsf1a*, or *Tnfrsf1b* between BTBR *ob/ob* mice and their respective WT controls (Fig. 6a, b, and c). In contrast, females WT exhibited increased MCP-1 gene expression (*Ccl2*) when compared to males WT, whereas females BTBR *ob/ob* showed reduced expression relative to females WT and expression similar to males BTBR *ob/ob* (Fig. 6d). Despite these changes, there was no difference in gene expression of the MCP-1 receptor (*Ccr2*) in tubular fractions among groups (Fig. 6e). Finally, increased transcript levels of *Nfkb1* in tubular fraction was detected only in male BTBR *ob/ob* mice compared to their respective WT controls (Fig. 6f). Notably, MCP-1 gene expression appears to be sex-dependent under basal conditions, whereas upregulation of *Nfkb1* transcript levels depend on both sex and metabolic condition, as it was observed only in obese males. The expression of *Lcn2*, which encodes neutrophil gelatinase-associated lipocalin (NGAL), is increased only in the tubules of male BTBR *ob/ob* mice (Fig. 6g), whereas the expression of *Havcr2*, which encodes kidney injury molecule 1 (KIM-1) is not differently expressed in male groups, but is surprisingly decreased in BTBR ob/ob female (Fig. 6h).

Taken together, these data indicate sex-dependent differences in gene expression of inflammatory and injury markers in tubular-enriched fractions in the BTBR *ob/ob* model.

### MCP-1 gene expression is increased in glomerular fractions of BTBR *ob/ob* mice, independent of sex

**Fig. 7:**
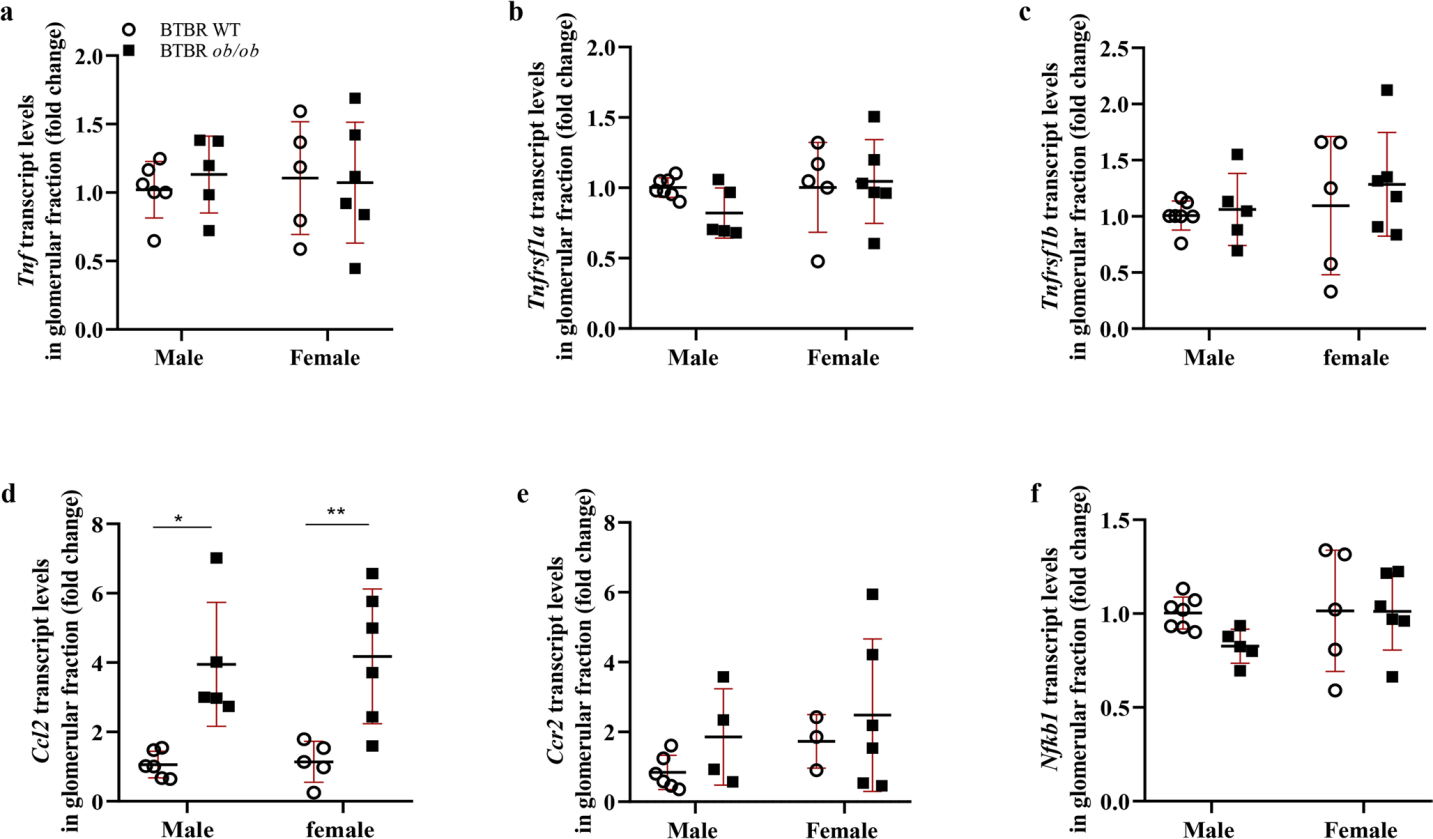
Inflammatory gene expression profile in the glomerular-enriched fraction of the BTBR *ob/ob* model. Images **A–F** show glomeruar transcript levels of *Tnf* (TNF-α), *Tnfrsf1a* (TNFR1), *Tnfrsf1b* (TNFR2), *Ccl2* (MCP-1), *Ccr2* (MCP-1 receptor), and *Nfkb1* (NFKB1), respectively, all expressed as fold change. P values of two-way ANOVA followed by Tukey’s multiple comparisons test are as follows: *p < 0.05, and **p < 0.01. Data are presented as mean ± SD (*n* = 3–7)

Subsequently, the expression of inflammatory genes in glomerular fractions was analyzed. The results indicate that there was no difference in the transcript levels of *Tnf*, *Tnfrsf1a*, or *Tnfrsf1b* in the glomerular fraction of male and female obese mice when compared to their respective controls (Fig. 7a, b, and c). However, BTBR *ob/ob* mice, regardless of sex, showed increased MCP-1 gene expression (*Ccl2*) in glomerular fractions compared to the WT controls (Fig. 7d). In addition, no significant alterations were observed in the transcript levels of *Ccr2* or *Nfkb1* in any of the studied groups (Fig. 7e and f). Collectively, these data show that, in this experimental model, hyperglycemia and obesity, independent of sex, are associated with increased *Ccl2* transcript levels specifically within the glomerular compartment, supporting the presence of a compartmentalized glomerular inflammatory response involving the MCP-1 pathway. Moreover, MCP-1-mediated macrophage recruitment may occur independently of detectable TNF-α/TNFR1 activation.

## Discussion

Diabetic kidney disease is a major complication of T2DM and represents a significant global health burden, given its strong contribution to chronic CKD and ESRD [26]. Despite growing interest in the role of biological sex in DKD progression, evidence remains inconsistent, with reports suggesting sex-dependent differences in renal outcomes but limited mechanistic knowledge. In this context, inflammation has been widely implicated as a contributor to renal injury in DKD [27]. However, whether sex modulates inflammation, including the understanding about specific renal compartments, remains insufficiently characterized, particularly in the BTBR *ob/ob* model. Albuminuria levels were detectable in obese males and females; however, sex-dependent differences were observed in podocyte-related gene and protein expression. In addition, the inflammatory profile assessed in this study was predominantly localized to the glomerular compartment in obese mice, with no detectable changes at the whole-kidney level, regardless of sex. These findings highlight the importance of considering compartment-specific analyses in DKD to improve the understanding of disease progression and support therapeutic strategies.

The results showed that obese males and females exhibited comparable body weight; however, females presented lower blood glucose levels under the same metabolic condition. This difference may reflect sex-dependent variations in insulin sensitivity and glucose homeostasis. In line with this, clinical studies indicate that females of reproductive age exhibit greater insulin sensitivity, enhanced insulin secretory capacity, and improved glucose tolerance compared to males, despite a higher proportion of body fat [28, 29]. These differences could be associated with greater metabolic flexibility in females, including increased mitochondrial activity-to-glycolysis ratio in some tissues [30]. Moreover, females appear to exhibit enhanced glucose uptake and utilization in insulin-responsive tissues, such as adipose tissue and skeletal muscle, potentially due to increased expression of glucose transporters and metabolic enzymes [31]. Together, these evidences suggest a more efficient metabolic adaptation in females, which may explain the lower glycemic levels observed in this study. However, a potential contribution of renal glucose metabolism cannot be excluded, given the role of the kidney in systemic glucose handling [32].

The mice used in this study were 16 weeks old, a stage at which DKD is considered established and progressive, as previously described by Hudkins et al. [33]. In fact, obese and diabetic mice exhibited increased albumin excretion regardless of sex, although this increase was more pronounced in males than females. This results were also previously observed by our group [34]. Albuminuria is associated with alterations in the glomerular filtration barrier, which is composed of the endothelial layer, the glomerular basement membrane (GBM), and the podocyte foot processes^35^. Together, these structures confer size and charge selectivity, preventing the passage of most plasma proteins, including albumin [35]. Podocytes are specialized cells that surround the glomerular capillaries, and their extensions form interdigitating foot processes connected by a specialized cell–cell junction known as the slit diaphragm [36]. Nephrin is a key structural component of this diaphragm, directly contributing to size selectivity of the filtration barrier. Thought its cellular domain, nephrin interacts with adaptor proteins that connect it to the actin cytoskeleton, coordinating signaling pathways essential for maintaining podocyte structure [36]. Loss or dysfunction of nephrin disrupts the slit diaphragm and leads to proteinuria [37], and urinary nephrin levels have been proposed as markers of podocyte injury and glomerular disease [38]. In the present study, despite the presence of albuminuria, no significant changes were observed in nephrin levels in renal tissue or in urinary nephrin excretion. Nevertheless, urinary nephrin excretion tended to increase, reaching levels approximately 12-fold higher than those of controls, which may still be biologically relevant. These findings suggest that additional mechanisms may contribute to albumin leakage in this model, including increased intraglomerular pressure, endothelial or vascular injury, structural alterations in the GBM, or even tubular injury. Notably, nephrin gene expression appeared to be sex-dependent at the basal level, with females exhibiting higher expression levels than males, regardless of metabolic condition. Similar observations have been reported in other models of renal injury, such as nephrectomy, in which females display higher basal expression of nephrin and other podocyte-related genes [39]. This pattern may suggest a more robust podocyte reserve in females, potentially conferring greater protection against progressive renal injury, although this remains to be further investigated.

Podocin is an integral membrane protein associated with lipid raft microdomains of the glomerular slit diaphragm, where it acts as a scaffold protein that binds to the cytoplasmic domain of nephrin, thereby contributing to the selectivity of the glomerular filtration barrier [40]. Mutations in NPHS2 gene, as well as defects in podocin trafficking or localization, disrupt slit diaphragm integrity and are strongly associated with proteinuria [41]. In the present study, only obese females exhibited increased podocin mRNA levels accompanied by reduced protein content compared to WT females and obese males, suggesting an interaction between sex and metabolic condition in podocin regulation. This pattern suggests disturbances in post-transcriptional processes, such as impaired protein folding, trafficking, or enhanced degradation, with the elevated transcript levels potentially representing a compensatory response. Notably, this reduction in podocin protein did not result in a corresponding increase in albuminuria in obese females relative to obese males, indicating that albumin excretion in this model may be driven by additional mechanisms as described, which were not explored in the present study. Previous study from our group also showed a decline in WT1-positive cells in both male and female obese mice, supporting the disruption of the glomerular filtration barrier [34].

To investigate the role of inflammation in DKD progression and the potential influence of sex, inflammatory markers were evaluated. No significant differences were observed in the protein levels of TNF-α and its receptors, as well as MCP-1, in total renal homogenates. Consistently, no increase in macrophage accumulation was detected in kidney sections. These findings suggest that, within the parameters assessed, BTBR *ob/ob* mice at 16 weeks of age do not exhibit detectable renal inflammation when the kidney is evaluated as a whole. However, a compartment-specific assessment revealed an increased presence of macrophages within the glomeruli of obese male mice, whereas females showed a strong trend toward increased macrophage infiltration, reaching levels approximately 92% higher than those of females WT. Evidence from studies in patients with T2DM and DKD [42], as well as in experimental models including streptozotocin-induced [43] and db/db mice [44], indicate that macrophage infiltration extends beyond the glomeruli to the tubulointerstitial compartment. Therefore, the predominance of these cells in glomeruli observed in this study may reflect an earlier stage of DKD, as glomeruli are among the first compartments affected by hyperglycemia, with initial immune cell recruitment occurring within the glomerular tuft. Tubulointerstitial inflammation may develop at later stages or remain less pronounced in this model. Moreover, an integrated axis linking hyperglycemia, inflammation, and glomerular injury is suggested, as positive correlations were observed between glomerular macrophage infiltration and blood glucose levels, nephrin excretion, and the ACR. Together, these findings indicate that inflammatory responses in BTBR *ob/ob* mice at 16 weeks of age are compartmentalized, with early changes predominantly localized to the glomerular compartment and associated with glomerular dysfunction. Future studies in older mice would be of interest to determine whether macrophage infiltration and structural lesions extend to the tubulointerstitial compartment at more advanced stages of DKD.

Based on these findings, it became essential to investigate the inflammatory profile of these mice in a compartment-specific manner, focusing on glomerular and tubular regions. In line with the previous observations, increased MCP-1 gene expression was detected in the glomerular compartment of obese mice, independent of sex. This pattern has also been suggested in other DKD models, including streptozotocin-induced rodents [45, 46] and db/db mice [47]. MCP-1 is a potent chemoattractant for inflammatory cells and is secreted by various cell types, including endothelial, epithelial, and mesangial cells, in response to inflammatory stimuli [48]. It binds to its receptor CCR2, which is primarily expressed on monocytes, triggering intracellular signaling pathways that promote cytoskeletal rearrangement and cell migration toward sites of inflammation [49]. Following transmigration across the endothelium, these cells differentiate into macrophages, which can further amplify inflammation through the release of proinflammatory cytokines such as IL-1β, IL-6, and TNF-α [49]. It is plausible that the MCP-1/CCR2 axis contributes to macrophage infiltration in the glomerular compartment of the BTBR *ob/ob* model with 16 weeks of age, particularly in male mice, despite the absence of changes in glomerular gene expression of TNF-α, its receptors, or CCR2. This interpretation is supported by the increased glomerular *Ccl2* gene expression and macrophage accumulation, suggesting activation of a compartmentalized inflammatory signaling pathway that may occur independently of detectable transcriptional changes in TNF-α-related genes.

In the tubular compartment, female WT mice exhibited higher MCP-1 gene expression compared to WT males. This finding suggests a sex-dependent basal regulation of this chemokine in tubular segments, potentially influenced by sex hormones. Previous studies have shown that MCP-1 is also involved in non-inflammatory pathways, including metabolism [50], tissue remodeling [51], and bone biology [52], which may require different basal levels between males and females. The absence of upregulation in obese females may support an association between MCP-1 expression and metabolic regulation in renal tubules. Additionally, obese males showed increased *Nfkb1* gene expression in the tubular compartment compared to their WT counterparts, a change that was not observed in females, supporting the notion that inflammatory responsiveness in the tubular compartment may differ between sexes under metabolic stress. Additionally, male obese mice exhibited significantly increased *Lcn2* expression, which encodes NGAL, which is upregulated and secreted by renal tubular epithelial cells in response to cellular stress and damage. This finding suggests that obesity induces a more pronounced tubular injury response in males. In contrast, the expression of the tubular injury marker *Havcr1* (which encodes KIM-1) was significantly reduced in female obese mice, indicating, again, a distinct sex-dependent pattern of renal injury. These observations are consistent with the findings reported by Kanoo et al. [53], who also demonstrated increased *Lcn2* expression in obese male mice together with lower *Havcr1* expression in obese females. Collectively, these results further support the existence of sexual dimorphism in the renal response to obesity, with males exhibiting a greater susceptibility to tubular stress and injury, whereas females appear to maintain a relatively protected phenotype.

A limitation of the present study is the lack of blood pressure analyses. Previous studies in the literature, however, have demonstrated that BTBR *ob/ob* mice develop progressive kidney disease in the absence of elevated blood pressure. Hudkins et al. (2010) [33] in fact reported lower blood pressure values in obese mice compared with wild-type controls. Also, more recently, Kanoo et al. (2024) [53] confirmed the same result. In that study, additional genetic reduction of nitric oxide bioavailability was used to develop hypertension. Taken together, these studies suggest that the renal injury observed in BTBR *ob/ob* mice occurs largely independently of elevated blood pressure.

Another limitation is that the present study does not establish a causal role for macrophages in the development of diabetic kidney disease. While increased glomerular macrophage accumulation was associated with markers of glomerular injury, including albuminuria and nephrin excretion, these findings are correlative in nature. Therefore, it remains unclear whether macrophages directly contribute to disease progression or represent a secondary response to ongoing tissue injury. Future studies employing macrophage depletion strategies, or targeted manipulation of macrophage will be necessary to determine the specific contribution of these cells to the initiation and progression of kidney injury in BTBR *ob/ob* mice.

In conclusion, kidney function was similarly impaired in male and female BTBR *ob/ob* mice, despite sex-dependent differences in podocyte-related gene and protein expression, indicating distinct molecular adaptations at the glomerular filtration barrier. Inflammatory responses were not detected at the whole-kidney level but were restricted to the glomerular compartment, where increased macrophage infiltration and MCP-1 gene expression were observed. In contrast, the tubular compartment exhibited a distinct, sex- and metabolism-dependent regulatory profile, highlighting the effect of sexual dimorphism in DKD. These findings are expected to contribute to a better understanding of DKD progression in an obesity context and may support the development of therapeutic strategies, reinforcing the importance of compartment-specific assessments that also take in account the biological sex.

## Electronic Supplementary Material

Below is the link to the electronic supplementary material.

Supplementary Material 1

High Resolution (EMF 119 KB)

## Data Availability

All data supporting the findings of this study are available within the paper and its Supplementary Information.
