# Supplementary figures and images for: Sex-dependent and compartment-specific macrophage accumulation associates with glomerular injury in BTBR ob/ob mice
Source: Pflugers Arch. 2026 Jul 28;478(8):69. doi: 10.1007/s00424-026-03195-8 (PMC13407935; doi:10.1007/s00424-026-03195-8)

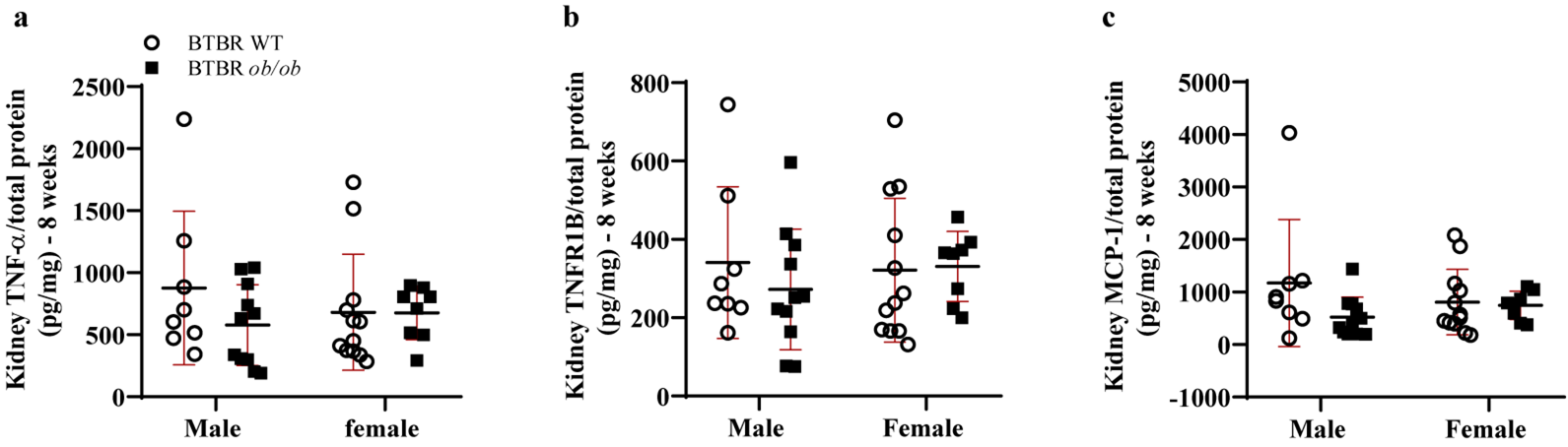

Supplement: Supplementary file 1 — Supplementary Material 1 [file 424_2026_3195_Fig8_ESM.png]
